# Effects of Dietary Deoxynivalenol on Growth Performance, Immunity, Reproductive Hormones, and Microbiome-Metabolome Responses in Immature Gilts

**DOI:** 10.3390/ani16111751

**Published:** 2026-06-05

**Authors:** Zaishan Li, Xiaoxia Hu, Helong Feng, Haiqing Sun, Jiajian Tan, Teng Yu, Zhengdan Lin, Guofu Cheng, Pin Chen

**Affiliations:** 1 College of Veterinary Medicine, Huazhong Agricultural University, Wuhan 430070, China; 2021302010126@webmail.hzau.edu.cn (Z.L.); lzd10152@webmail.hzau.edu.cn (Z.L.); 2 Guangxi Yangxiang Group Co., Ltd., Guigang 537100, China; 15977584352@163.com (X.H.); sunhaiqing1985110@163.com (H.S.); tjjggyx@sina.com (J.T.); yxyt2016@163.com (T.Y.); 3 Institute of Animal Husbandry and Veterinary Sciences, Hubei Academy of Agricultural Sciences, Wuhan 430064, China; fenghelong_clp@163.com

**Keywords:** deoxynivalenol, gilts, growth performance, microbiome-metabolome response

## Abstract

We evaluated the effects of different levels of DON (LD 441 and HD 1223 μg DON/kg in diet) on the growth performance, immunity, reproductive hormones, and intestinal health of immature gilts. No significant differences were observed in average daily gain, average daily feed intake, or feed to gain ratio between the LD group and the HD group (*p* > 0.05). The red blood cell count and hematocrit were higher in the LD group compared with the HD group on d 21 (*p* < 0.05). The gamma-glutamyl transferase activity in the LD group on d 1, 21, 28, 35, and 42 was higher (*p* < 0.05) compared with the HD group. The aspartate aminotransferase, total antioxidant capacity, and lactic dehydrogenase levels on d 35 were higher in the LD group than those in the HD group (*p* < 0.05). On d 35, the levels of interleukin 1β, interleukin-4, interleukin-10, tumor necrosis factor-α, and interferon-γ in LD were higher than those in the HD group (*p* < 0.05). The levels of immunoglobulin A, immunoglobulin M, immunoglobulin G, and complement 4 on d 35 were higher in the LD group compared with those in the HD group (*p* < 0.05). The gonadotrophin-releasing hormone, luteotrophic hormone, follicle-stimulating hormone, or estradiol did not differ between LD and HD groups throughout the experiment (*p* > 0.05). For fecal microbiota, *Streptococcus* in the HD group was reduced compared with the LD group (*p* < 0.05). In summary, feeding diets contaminated with 1223 μg DON/kg exerted adverse effects on serum profiles of gilts but did not affect their growth performance or reproductive hormones in the present study.

## Simple Summary

The limited level of DON and its influence on immature gilts were unknown. Therefore, we evaluated the effects of different levels of DON (LD 441 and HD 1223 μg DON/kg in diet) on the growth performance, immunity, reproductive hormones, and intestinal health of immature gilts. A total of 84 immature gilts (Landrace × Yorkshire) were randomly divided into two groups with six replicates (pens) per group and seven immature gilts per replicate based on the concentration of DON in diets. Dietary treatments did not affect growth performance. High DON caused some negative effects on serum profiles in immature gilts. Dietary treatments did not affect serum hormones. In summary, feeding diets contaminated with 1223 μg DON/kg exerted some adverse effects on serum profiles of gilts, but did not affect their growth performance or reproductive hormones in the present study.

## 1. Introduction

Various feedstuffs are susceptible to being contaminated with mycotoxins, secondary metabolites produced by molds, which can cause adverse physiological effects on pigs [1]. The common mycotoxins that affect pig production include deoxynivalenol (DON), aflatoxin B_1_, and fumonisins, and these toxins naturally contaminate common feed ingredients and complete diets for pigs [2,3].

Commonly referred to as “vomitoxin”, DON is a prevalent contaminant found in food crops globally. It is a worldwide prevalent Type B trichothecene produced primarily by *Fusarium graminearum* and *Fusarium culmorum* [4]. Production of DON is possible under regular climatic conditions, yet it takes place primarily in cool and humid environments [5,6]. It could reduce feed intake of pigs, with subsequent adverse effects including digestive disorder, organ dysfunction, immunosuppression, and decreased growth performance [7,8,9]. Furthermore, DON could impair reproductive and developmental function, damage the gastrointestinal tract, and induce apoptosis, oxidative stress, and genotoxicity of porcine oocytes [10,11].

The production of DON may cause substantial economic losses and severe damage to the pig industry [2]. The pig feed is composed of corn, barley, distillers dried grains with solubles (DDGS), soybean meal, and other raw materials [3]. Cereal raw materials such as corn and DDGS are prone to DON contamination, which may consequently lead to excessive DON levels in the final feed products. Previous studies have consistently demonstrated that diets contaminated with DON exert detrimental effects on weanling pigs [4,12], growing-finishing pigs [13,14], immature gilts [15], and first-parity sows [16].

The U.S. Food and Drug Administration and the Ministry of Agriculture and Rural Affairs of China had both set a maximum limit of 1000 µg/kg for DON in grains (such as corn, wheat, etc.), grain products, and final feed for pigs. In addition, the European limit was 900 µg/kg DON in final feed for pigs. The level of DON in diets of weanling pigs, sows, and immature gilts was controlled below 500 µg/kg in the actual production applications of China. The price of corn (the main component of pig diet) was increased by 1.5–3.0% under this level, and thus increased the cost of pig farming. It was reported that DON-contaminated diets could affect the immunity in weanling pigs [4,17,18]. The immature gilts are one of the breeding pig sources and play a significant role in ensuring the reproductive capacity in the pig farms. However, the limited level of DON and its influence on immature gilts were unknown. Here, this study evaluated the effects of different levels of DON (low-dose: 441 µg/kg in diets, high-dose: 1223 µg/kg in diets) on the growth performance, immunity, reproductive hormones, and intestinal health of immature gilts. The present study will have important clinical guiding significance for actual pig production.

## 2. Materials and Methods

### 2.1. Ethics Statement

Experimental procedures and animal care used in this study were in accordance with the guidelines of the care and use of laboratory animals issued by the Animal Care Committee of Huazhong Agricultural University (approval number: HZAUMO-2023–0289).

### 2.2. Animals and Diets

A total of 84 immature gilts (Landrace × Yorkshire) were from Guangxi Yangxiang Group Co., Ltd. (Guigang, China), at the age of 131 days with an average initial BW of 82.60 ± 1.12 kg. All immature gilts freely have access to water and diets in the experiment, and were housed in a facility with slatted plastic flooring and a mechanical ventilation system. The immature gilts in the LD and HD groups were vaccinated with commercial breeding vaccines.

Before the experiment, the normal, naturally contaminated corn and distillers dried grains with soluble (DDGS) samples were collected, and the aflatoxin (AF), ochratoxin (OA), DON, and zearalenone (ZEA) were determined by High Performance Liquid Chromatography (HPLC) according to previous studies [15]. The detection limits of AF, OA, ZEA, and DON concentration were 0.1 μg/kg, 0.5 μg/kg, 1.5 μg/kg, and 20 μg/kg, respectively. The DON of corn, barley, soybean meal, and DDGS in the LD group diet was 480, 200, 1100, and 200 μg/kg, while the DON of corn, barley, soybean meal, and DDGS in the HD group diet was 1860, 200, 1100, and 200 μg/kg, respectively. The diets were formulated based on the determined mycotoxin content of corn and DDGS. The dry matter, crude protein, ether extract, crude fiber, total ash, calcium, and phosphorus in the experiment diets were determined based on the standard procedures of the AOAC [19]. The dry matter of the diets was determined after drying for 24 h at 103 °C. Crude protein content was determined by using a Kjeltec 2300 Analyzer (Foss Tecator AB, Hoeganaes, Sweden). Total ash was determined after ignition of a weighed sample in a muffle furnace (Nabertherm, Bremen, Germany) at 550 °C for 6 h. The ash was then digested in aqua regia (HCl/HNO_3_ mixture), and the solution was used for calcium (Ca) and phosphorus (P) determination. Ca concentration was determined using an atomic absorption spectrophotometer (Varian’50, Varian, Palo Alto, CA, USA), and the concentration of P was determined spectrophotometrically (NanoDrop 2000c, Thermo Scientific, MA, USA) using the method described previously. The amino acids of all the experimental diets were analyzed by an amino acid analyzer (Biochrom 20, Pharmacia Biotech, Cambridge, UK).

### 2.3. Experimental Design

All immature gilts were randomly divided into 2 groups based on the concentration of DON in diets, with 6 replicates (pens) per group and 7 immature gilts per replicate. The groups include low-dose DON (LD, 441 μg/kg) and high-dose DON (HD, 1223 μg/kg). The diets of immature gilts were formulated according to National Research Council (NRC) requirements (Table 1) [20]. On days 15, 30, and 45 of the experiment, the immature gilts were weighed, and the diet consumption per pen was also determined. The average daily gain (ADG), average daily feed intake (ADFI), and feed-to-gain ratio (F/G) were calculated. The mortality was recorded during the experiment period.

### 2.4. Sample Collection and Treatment

The blood samples of 7 immature gilts per replicate (pen) were randomly collected on days 1, 7, 14, 21, 28, 35, and 42 of the experiment, and stored at 4 °C. The levels of white blood cell (WBC), lymphocyte, granulocyte, intermediate cell (MID), red blood cell (RBC), hemoglobin (HGB), hematocrit (HCT), mean corpuscular volume (MCV), mean cell hemoglobin (MCH), and platelet (PLT) were determined by an automatic blood biochemical analyzer (PE-3070VET, Prokan Corp., Baoan, Shenzhen, China).

The serum malondialdehyde (MDA), catalase (CAT), endotoxin (ET), total antioxidant capacity (T-AOC), lactic dehydrogenase (LDH), diamine oxidase (DAO), alanine transaminase (ALT), aspartate aminotransferase (AST), gamma-glutamyl transferase (GGT), immunoglobulin A (IgA), immunoglobulin M (IgM), immunoglobulin G (IgG), complement 3 (C3), complement 4 (C4), interleukin 1β (IL-1β), interleukin-4 (IL-4), interleukin-4 (IL-10), tumor necrosis factor-α (TNF-α) and interferon-γ (IFN-γ) were determined using commercial ELISA kits (Jiancheng Biotechnology Institute, Nanjing, China). The serum estradiol (E2), luteotrophic hormone (LH), follicle-stimulating hormone (FSH), and gonadotrophin-releasing hormone (GnRH) were detected by the commercial ELISA kits (Jiancheng Biotechnology Institute, Nanjing, China).

### 2.5. Analysis of Microbiome-Metabolome

On days 1, 7, 21, 35, and 42 of the experiment, fecal samples were collected via rectal massage from all pigs per pen, then pooled, and transported to the lab for the analysis of Microbiome-Metabolome. Briefly, total genomic DNA of bacteria in the feces was extracted using the commercial DNA extraction kit (Ultra clean fecal DNA isolation kit, Solarbio Co., Ltd., Beijing, China). The concentration of DNA was measured by the Nano-Drop 1000 spectrophotometer (Thermo Scientific Inc., Wilmington, DE, USA). The 16S rRNA gene sequencing was performed using Illumina Miseq PE250 sequencer (Illumina, Inc., San Diego, CA, USA), and the low-quality sequences were removed using Mothur software. The operational taxonomic units (OTU) picking with 97% similarity cut-off was compiled based on default parameters, and taxonomic classification was performed using the OTU database. The microbial alpha-diversity indices (including Chao, Ace, Shannon, and Simpson) were calculated with the Mothur program (http://www.mothur.org) and Rarefactuin software 1.39.5. The data of Microbiome-Metabolome have been deposited in the NCBI.

### 2.6. Histopathological Analysis

At 45 days of the experiment, all pigs were slaughtered, and the left stomach, liver, spleen, lymph node, and duodenum from each group were collected by trained personnel and fixed in 10% neutral formalin. The fixed tissues were trimmed, paraffin-embedded, and sectioned (4 μm). Thin sections (4 μm) were sliced and mounted onto slides and then stained with Hematoxylin and Eosin (H&E) for histopathological examination using an Olympus optical microscope (Olympus microscope, Olympus Corporation, Tokyo, Japan).

### 2.7. Statistical Analysis

Data were analyzed using IBM SPSS Statistics (version 31.0.1.0, IBM Corp., Armonk, NY, USA). For parameters measured at multiple time points and other time-segmented growth performance data, a two-way repeated-measures linear mixed model was used with toxin dose, time, and their interaction as fixed effects, and individual replicate as a random effect. Pairwise comparisons were performed with Bonferroni correction. For 1–45 d of cumulative data, independent samples *t*-tests were used.

All data are presented as mean ± SEM. Statistical significance was set at *p* < 0.05. In figures, asterisks denote significance levels as follows: * *p* < 0.05, ** *p* < 0.01, *** *p* < 0.001. Graphs were prepared using GraphPad Prism 9.0 (GraphPad Software, San Diego, CA, USA).

## 3. Results

### 3.1. The Detection of Mycotoxin Concentrations in Diets

The DON of corn, barley, soybean meal, and DDGS in the LD group diet was 480, 200, 1100, and 200 μg/kg, while the DON of corn, barley, soybean meal, and DDGS in the HD group diet was 1860, 200, 1100, and 200 μg/kg, respectively. The concentrations of DON and ZEA in the diets were 441 μg DON/kg diet in low-dose groups, as well as 1223 μg DON/kg diet in high-dose groups, respectively. Other mycotoxins did not exceed the limit in the diets.

### 3.2. Analysis of Growth Performance

**Figure 1:**
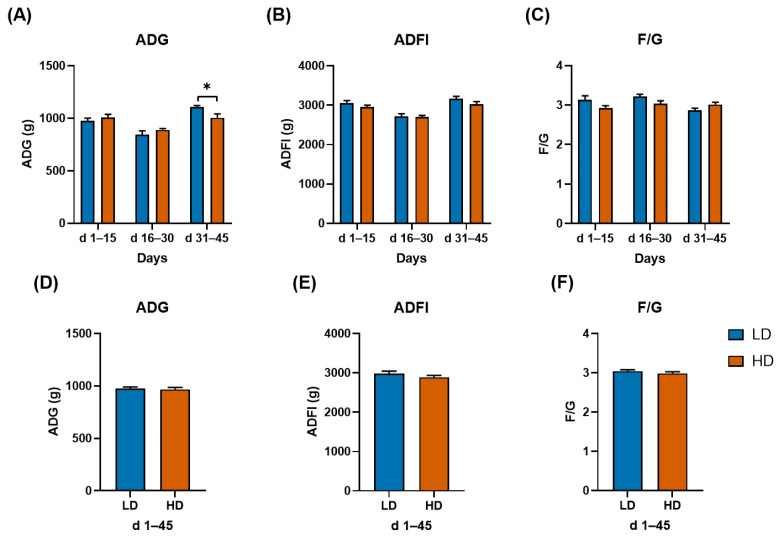
Effects of different levels of DON on growth performance in immature gilts ^1,2^. ^1^ Means represent 6 replications per treatment (*n* = 6/group). ^2^ LD, low-dose 441 μg DON/kg diet; HD, high-dose 1223 μg DON/kg diet; ADG, average daily gain; ADFI, average daily feed intake; F/G, feed to gain ratio. (**A**) effects of dietary treatments on ADG during d 1–15, 16-30 and 31-45; (**B**) effects of dietary treatments on ADFI during d 1–15, 16-30 and 31-45; (**C**) effects of dietary treatments on F/G during d 1–15, 16-30 and 31-45; (**D**) effects of dietary treatments on ADG during d 1-45; (**E**) effects of dietary treatments on ADFI during d 1-45; (**F**) effects of dietary treatments on F/G during d 1-45. * means a probability level of *p* < 0.05.

The ADG, ADFI, and F/G were calculated at different time points. As shown in Figure 1, the ADG of immature gilts from the LD group was higher than that from the HD group during d 31–45 (*p* = 0.013), while dietary treatments did not affect ADG during d 1–15, d 16–30, or d 1–45 (*p* > 0.05). There were no differences in the ADFI or F/G of immature gilts throughout the experiment between LD and HD groups (*p* > 0.05).

### 3.3. Detection of Blood Routine Indexes

**Figure 2:**
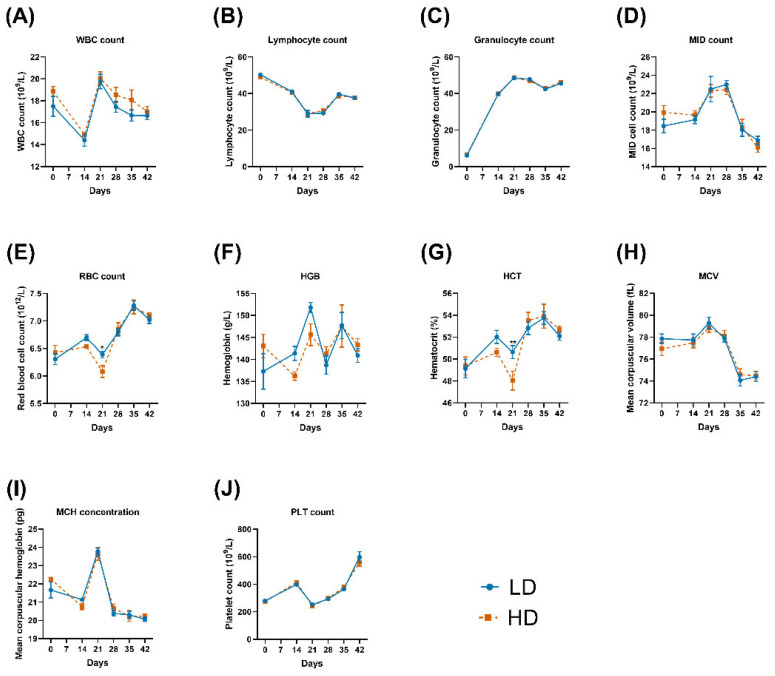
Effects of different levels of DON on blood profiles in immature gilts ^1,2^. ^1^ Means represent 42 replications per treatment (*n* = 42/group). ^2^ LD, low-dose 441 μg DON/kg diet; HD, high-dose 1223 μg DON/kg diet; WBC, white blood cell; MID, intermediate cells (monocytes, eosinophils, and basophils); RBC, red blood cell; HGB, hemoglobin; HCT, hematocrit; MCV, mean corpuscular volume; MCH, mean cell hemoglobin; PLT, platelet. (**A**) effects of dietary treatments on WBC count; (**B**) effects of dietary treatments on lymphocyte count; (**C**) effects of dietary treatments on granulocyte count; (**D**) effects of dietary treatments on MID count; (**E**) effects of dietary treatments on RBC count; (**F**) effects of dietary treatments on HGB; (**G**) effects of dietary treatments on HCT; (**H**) effects of dietary treatments on MCV; (**I**) effects of dietary treatments on MCH concentration; (**J**) effects of dietary treatments on PLT count. * means a probability level of *p* < 0.05 and ** means a probability level of *p* < 0.01.

As shown in Figure 2, no differences were observed in WBC, lymphocyte, granulocyte, MID, HGB, MCV, MCH, or PLT among groups throughout the experiment (*p* > 0.05). The RBC (*p* = 0.013) count and HCT (*p* = 0.009) were higher in the LD group compared with the HD group on d 21 (*p* < 0.05).

### 3.4. Detection of Antioxidant Stress

**Figure 3:**
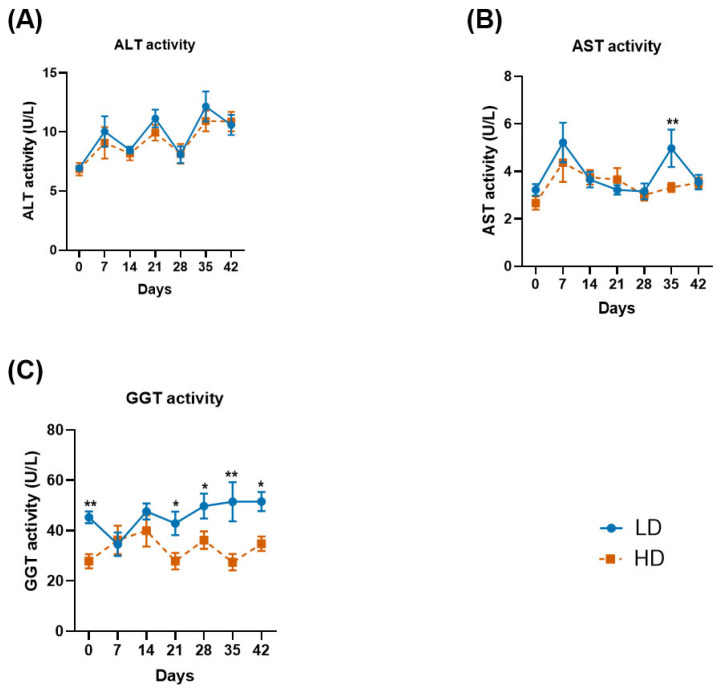
Effects of different levels of DON on serum liver function in immature gilts ^1,2^. ^1^ Means represent 42 replications per treatment (*n* = 42/group). ^2^ LD, low-dose 441 μg DON/kg diet; HD, high-dose 1223 μg DON/kg diet; ALP, alkaline phosphatase; DAO, diamine oxidase; ALT, alanine transaminase; AST, aspartate aminotransferase; GGT, gamma-glutamyltransferase. (**A**) effects of dietary treatments on ALT activity; (**B**) effects of dietary treatments on AST activity; (**C**) effects of dietary treatments on GGT activity. * means a probability level of *p* < 0.05 and ** means a probability level of *p* < 0.01.

In Figure 3, dietary treatments did not affect the levels of ALT activity in the entire experiment (*p* > 0.05). There were no differences in AST activity between LD and HD groups (*p* > 0.05); only the AST in the LD group was higher than that in the HD group on d 35 (*p* = 0.008). The GGT activity in the LD group on d 1, 21, 28, 35, and 42 was higher (*p* < 0.05) compared with that in the HD group without any effect on d 7 or 14 (*p* > 0.05).

**Figure 4:**
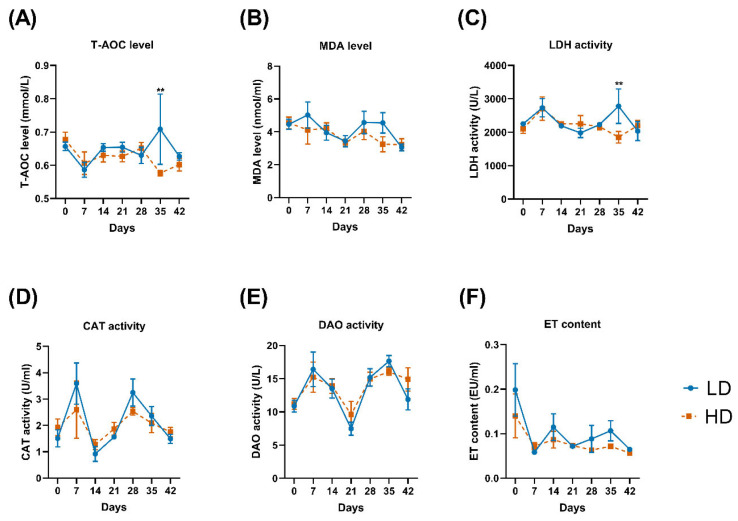
Effects of different levels of DON on serum antioxidant capacity in immature gilts ^1,2^. ^1^ Means represent 42 replications per treatment (*n* = 42/group). ^2^ LD, low-dose 441 μg DON/kg diet; HD, high-dose 1223 μg DON/kg diet; T-AOC, total antioxidant capacity; MDA, malondialdehyde; LDH, lactic dehydrogenase; CAT, catalase; DAO, diamine oxidase. (**A**) effects of dietary treatments on T-AOC level; (**B**) effects of dietary treatments on MDA level; (**C**) effects of dietary treat-ments on LDH activity; (**D**) effects of dietary treatments on CAT activity; (**E**) effects of dietary treatments on DAO activity; (**F**) effects of dietary treatments on ET count. ** means a probability level of *p* < 0.01.

In Figure 4, the levels of MDA, ET, CAT, and DAO did not differ between LD and HD groups (*p* > 0.05). The T-AOC (*p* = 0.005) and LDH (*p* = 0.003) levels on d 35 were higher in the LD group than those in the HD group (*p* < 0.05), while no differences were observed at other time points (*p* > 0.05).

### 3.5. Analysis of Immune Cytokines

**Figure 5:**
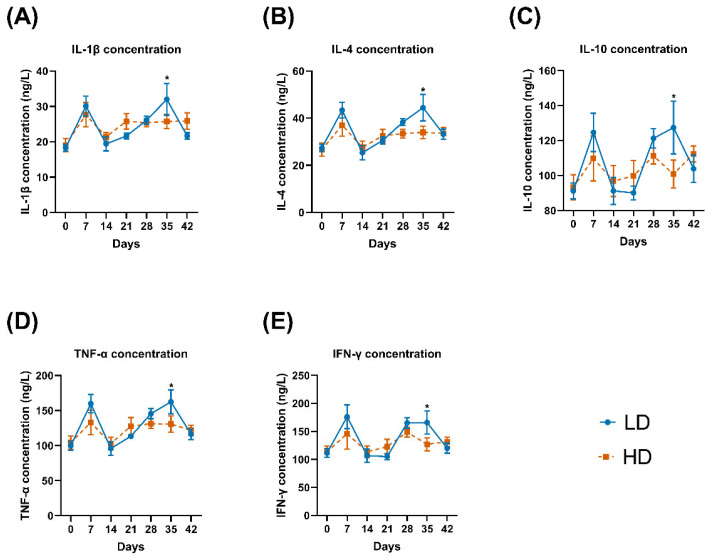
Effects of different levels of DON on serum inflammatory factors in immature gilts ^1,2^. ^1^ Means represent 42 replications per treatment (*n* = 42/group). ^2^ LD, low-dose 441 μg DON/kg diet; HD, high-dose 1223 μg DON/kg diet; IL-1β, interleukin 1β; IL-4, interleukin-4; IL-10, interleukin-4; TNF-α, tumor necrosis factor-α; IFN-γ, interferon-γ; ET, endotoxin. (**A**) effects of dietary treatments on IL-1β concentration; (**B**) effects of dietary treatments on IL-4 concentration; (**C**) effects of dietary treatments on IL-10 concentration; (**D**) effects of dietary treatments on TNF-α concentration; (**E**) effects of dietary treatments on IFN-γ concentration. * means a probability level of *p* < 0.05.

On d 35, the levels of IL-1β (*p* = 0.004), IL-4 (*p* = 0.012), IL-10 (*p* = 0.028), TNF-α (*p* = 0.039) and IFN-γ (*p* = 0.044) in the LD group were higher than those in the HD group (Figure 5, *p* < 0.05), but dietary treatments did not affect those immune cytokines at other time points (*p* > 0.05). No differences were observed in ET between LD and HD groups throughout the experiment (*p* > 0.05).

**Figure 6:**
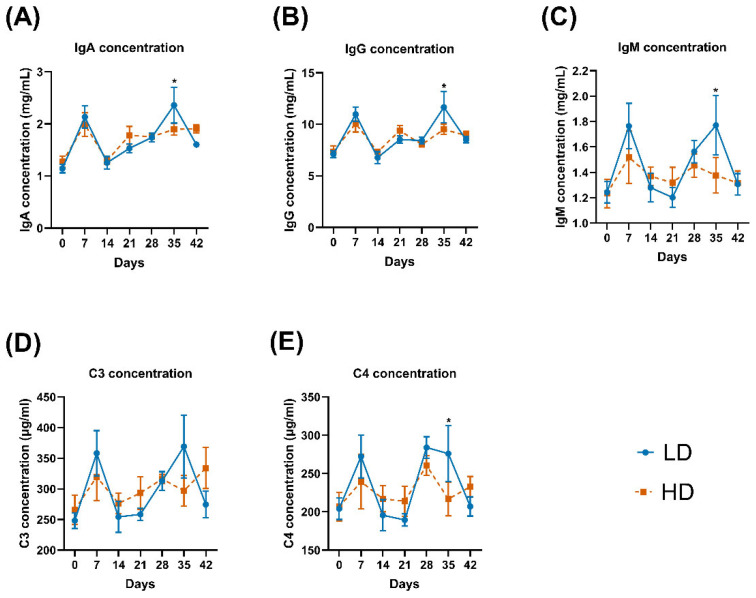
Effects of different levels of DON on serum immunity in immature gilts ^1,2^. ^1^ Means represent 42 replications per treatment (*n* = 42/group). ^2^ LD, low-dose 441 μg DON/kg diet; HD, high-dose 1223 μg DON/kg diet; IgA, immunoglobulin A; IgG, immunoglobulin G; IgM, immunoglobulin M; C3, complement 3; C4, complement 4. (**A**) effects of dietary treatments on IgA concentration; (**B**) effects of dietary treatments on IgG concentration; (**C**) effects of dietary treatments on IgM concentration; (**D**) effects of dietary treatments on C3 concentration; (**E**) effects of dietary treatments on C4 concentration. * means a probability level of *p* < 0.05.

In Figure 6, the levels of IgA (*p* = 0.029), IgM (*p* = 0.029), IgG (*p* = 0.016), and C4 (*p* = 0.044) on d 35 were higher in the LD group compared with those in the HD group (*p* < 0.05), while no differences were observed at other time points (*p* > 0.05). Dietary treatments did not influence the level of C3 throughout the experiment (*p* > 0.05).

### 3.6. Reproductive Hormones

**Figure 7:**
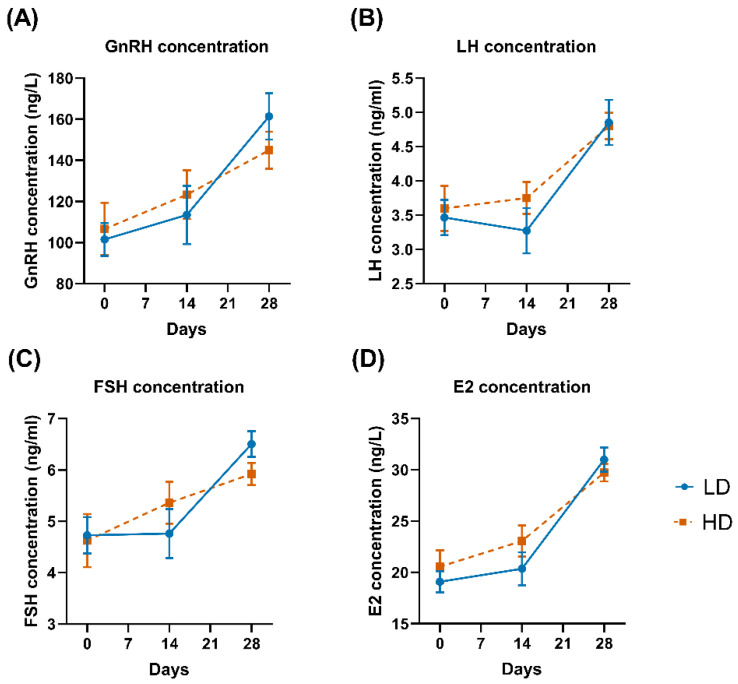
Effects of different levels of DON on serum reproductive hormones in immature gilts ^1,2^. ^1^ Means represent 42 replications per treatment (*n* = 42/group). ^2^ LD, low-dose 441 μg DON/kg diet; HD, high-dose 1223 μg DON/kg diet; GnRH, gonadotrophin releasing hormone; LH, luteotrophic hormone; FSH, follicle-stimulating hormone; E2, estradiol. (**A**) effects of dietary treatments on GnRH concentration; (**B**) effects of dietary treatments on LH concentration; (**C**) effects of dietary treatments on FSH concentration; (**D**) effects of dietary treatments on E2 concentration.

The results of reproductive hormones showed that GnRH, LH, FSH, or E2 did not differ between LD and HD groups in the whole experiment (Figure 7; *p* > 0.05).

### 3.7. Microbiome-Metabolome in Feces

**Figure 8:**
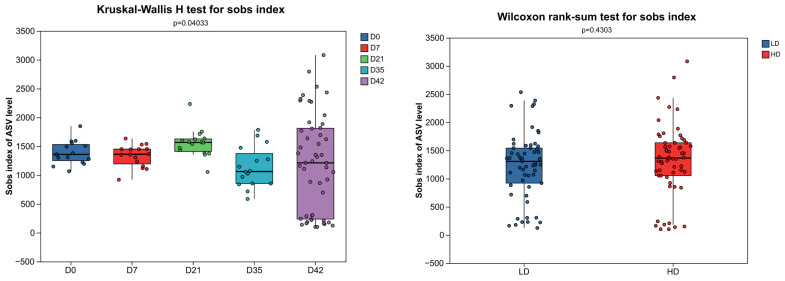
α-diversity analysis difference test at different periods (Post hoc test, *p* < 0.05, *n* = 16–64/group) ^1^. ^1^ LD, low-dose 441 μg DON/kg diet; HD, high-dose 1223 μg DON/kg diet (*n* = 64/group).

The Microbiome-Metabolomes in feces were analyzed at five time points (P1: day 1; P2: day 7; P3: day 21; P4: day 35; P5: day 42). As shown in Figure 8, the results of α-diversity showed that there were significant differences in Simpson and Shannon indices among the three time periods (P1 and P5; P2 and P5; P3 and P5; *p* < 0.05). There were also significant differences in the homogeneity and richness of fecal microorganisms sampled at different times (*p* < 0.05). There was no difference in the sobs index between LD and HD groups (*p* > 0.05).

**Figure 9:**
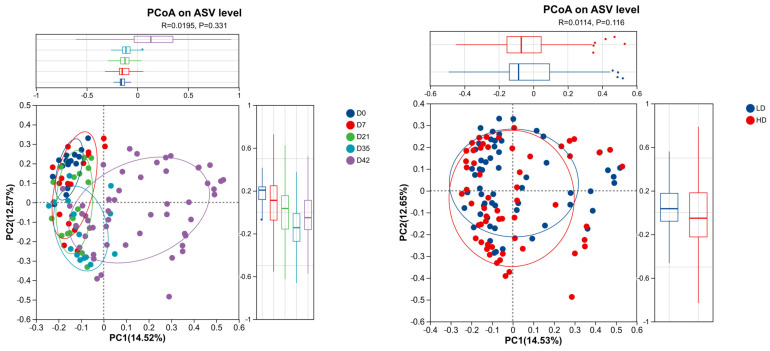
β-diversity analysis PCoA diagram at different periods (annosim test, *p* < 0.05, *n* = 16–64/group) ^1^. ^1^ LD, low-dose 441 μg DON/kg diet; HD, high-dose 1223 μg DON/kg diet (n = 64/group).

**Figure 10:**
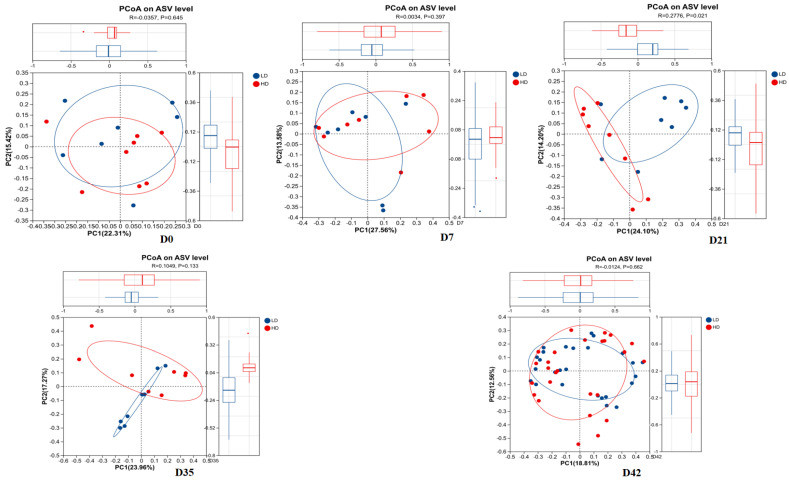
β–diversity analysis PCoA diagram (annosim test, *p* < 0.05, *n* = 8–32/group) ^1^. ^1^ LD, low-dose 441 μg DON/kg diet; HD, high-dose 1223 μg DON/kg diet.

There was no significant difference in the β-diversity of fecal microorganisms in the same group at different time points (Figure 9 and Figure 10; *p* > 0.05). The fecal microbiota β-diversity did not differ between LD and HD groups (Figure 9; *p* > 0.05).

**Figure 11:**
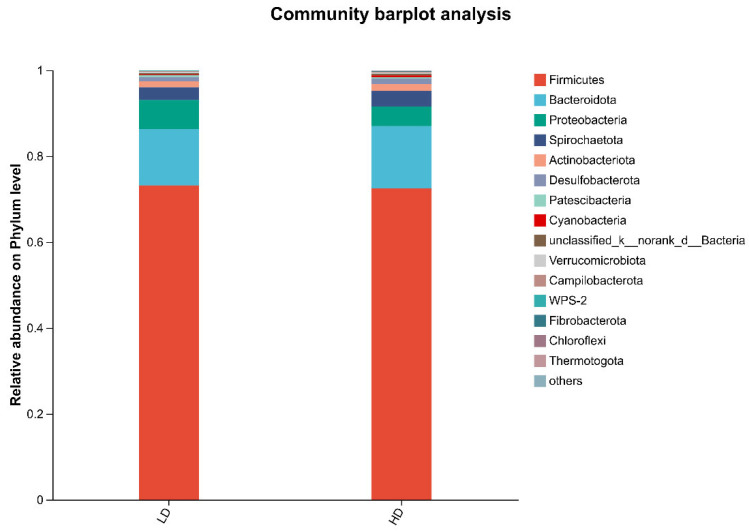
Composition of microbiological phylum level in sampled pigs at different periods ^1^ (*n* = 64/group). ^1^ LD, low-dose 441 μg DON/kg diet; HD, high-dose 1223 μg DON/kg diet.

**Figure 12:**
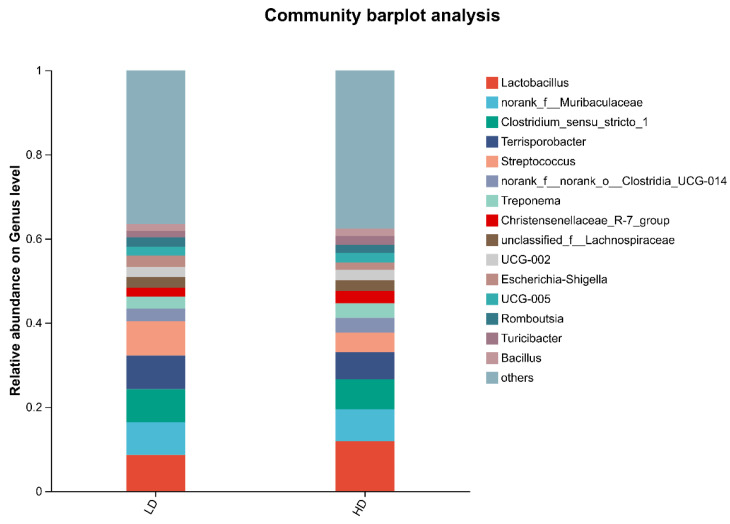
Composition of microbiological genus level in sampled pigs at different periods ^1^ (*n* = 64/group). ^1^ LD, low-dose 441 μg DON/kg diet; HD, high-dose 1223 μg DON/kg diet.

**Figure 13:**
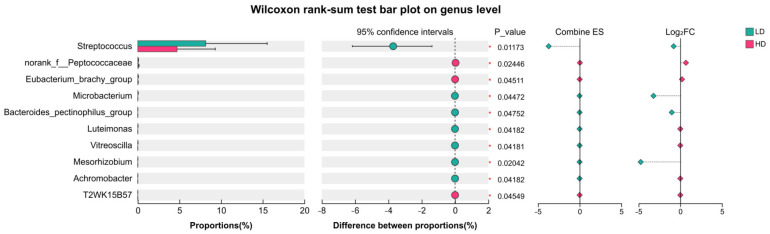
Bar charts of different species of sampled pigs at different periods ^1^ (n = 64/group). ^1^ LD, low-dose 441 μg DON/kg diet; HD, high-dose 1223 μg DON/kg diet. * means a probability level of *p* < 0.05.

**Figure 14:**
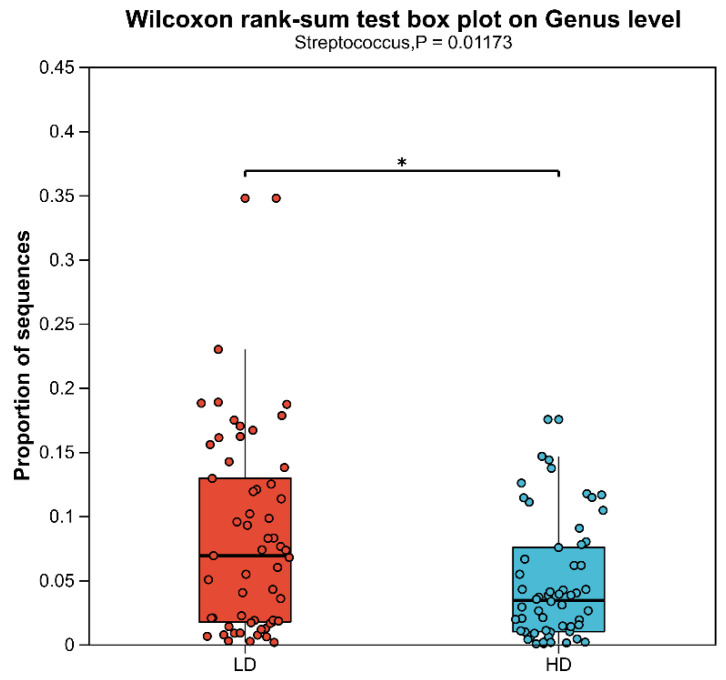
Bar charts of different species of sampled pigs at different periods ^1^ (*n* = 64/group). ^1^ LD, low-dose 441 μg DON/kg diet; HD, high-dose 1223 μg DON/kg diet. * means a probability level of *p* < 0.05.

The relative abundance ratio of the phylum levels from high to low was as follows: *Firmicutes*, *Bacteroidetes*, *Spirales*, *Actinomycetes*, *Proteobacteria*, etc. (Figure 11). The proportion of relative abundance of the genera was *Lactobacillus*, *Streptococcus*, *Clostridium*, *Treponema, Bacillus earthen*, etc., from high to low (Figure 12). There were no differences in the phylum levels (Table 2). However, the abundance of harmful bacteria *Streptococcus* in the HD group was significantly reduced than in the LD group (*p* < 0.05) (Table 3; Figure 13 and Figure 14).

### 3.8. Histopathological Analysis

**Figure 15:**
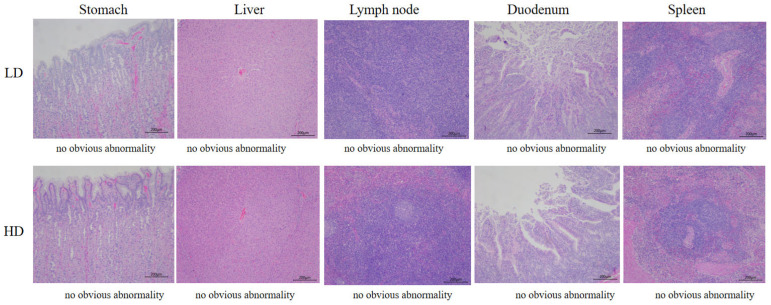
Effects of different levels of DON on histopathology in organs of immature gilts ^1^ (*n* = 6/group). ^1^ LD, low-dose 441 μg DON/kg diet; HD, high-dose 1223 μg DON/kg diet.

Histopathology of the stomach, liver, spleen, lymph node, and duodenum was evaluated at 45 days of the experiment. As shown in Figure 15, no histopathological damage was observed in the stomach, liver, spleen, lymph node, and duodenum among the groups. The results demonstrated that different doses of DON did not cause histopathological damage.

## 4. Discussion

The gilts were vulnerable to DON because their detoxification ability may be relatively low [5,21]. A 45-day exposure period in immature gilts (from approximately 131 days of age) represents a meaningful window during the pre-pubertal developmental phase. In this study, the effects of DON were evaluated on growth performance, serum profiles, hormones, histopathology, and microbiome-metabolome in immature gilts. The low-dose DON (441 μg DON/kg) was considered safe in the actual production applications (below 500 μg DON/kg with increased cost of diets), and the high-dose DON (1223 μg DON/kg) exceeded the limit set by the Ministry of Agriculture and Rural Affairs of China and the European Food Safety Authority.

Previous studies demonstrated that long-term exposure to DON might reduce feed intake, weight gain, and nutrient absorption efficiency in growing-finishing pigs [8,13,22]. We only observed the ADG difference during d 31–45, which might be due to the duration of exposure. However, the present study showed that the dose of DON did not affect the growth performance of immature gilts throughout the experiment. While significant differences were noted in the final experimental period, the lack of significant effects during the preceding stages led to no overall significant difference throughout the full trial duration. This discrepancy may be due to the relatively low DON level and short duration in the current study compared with other studies. Similarly, a recent study indicated that the diets containing 3590 or 5720 μg/kg DON decreased final BW, ADG, and ADFI in finishing pigs from 76.6 kg for 43 d, while the diets containing 1340 μg/kg DON did not affect growth performance [14]. Even the low-dose DON (600–900 μg/kg) did not influence growth performance in weaning pigs [13]. A consistent decrease in ADG and final BW was observed in growing-finishing pigs fed diets with 4800–6100 μg/kg DON [8,23]. Based on EFSA Panel on Contaminants in the Food Chain [24,25], for finishing pigs, a lowest observed adverse effect level (LOAEL) of 1600 μg/kg was identified. This was just consistent with the DON level in the present study. Therefore, it was supposed that immature gilts may be tolerant to low-dose DON (<1000 μg/kg).

Previous studies have demonstrated that DON may disrupt homeostasis and lead to some changes in serum profiles [25]. In the current study, the serum IL-1β, IL-4, IL-10, TNF-α, and IFN-γ were decreased in immature gilts fed 1223 μg/kg DON diets compared with those fed 441 μg/kg DON. This agreed with a previous study, which showed that feeding 1200–2000 μg/kg DON downregulated serum IL-1β, IL-8, and TNF-α in weaning pigs [26]. Feeding 2800 μg/kg DON also induced the expression of IL-1β, IL-2, IL-6, IL-12, and MIP-1β in the jejunum and induced the expression of TNF-α, IL-1β, and IL-6 in the ileum of pigs [27]. Several studies have indicated that 750 µg/kg DON increased TNF-α and IL-6 expressions in weaning pigs, while 3000 µg/kg DON enhanced transforming growth factor beta and IL-10 expressions [7].

A review demonstrated that feeding DON-contaminated diets increased serum IgA in pigs [28], which was consistent with the present results. It was found that the immature gilts fed 1223 μg/kg DON diets had higher serum IgA, IgM, IgG, and C4 than those fed 441 μg/kg DON diets on d 35 in the current study. Others also observed the increased serum IgA in weaning pigs fed DON-contaminated diets [21]. This may be due to the increasing procytokines production, which promoted the generation of IgA in pigs exposed to the low-dose DON [7,25]. The increased serum IL-1β might enhance differentiation of IgA-secreting B cells [15]. The serum immune indicators responded to DON earlier and more sensitively compared with growth performance in the current study. We speculate that the lack of significant differences in serum inflammatory factors and immune indicators on d 42 may be attributed to the possible adaptation of gilts to DON exposure after day 35. Nevertheless, further research is warranted to verify this hypothesis. Previous studies have documented that pigs may exhibit adaptive responses to chronic DON exposure. It is reported that hepatic transcriptomic responses to chronic dietary DON were most pronounced during the early phase of exposure, with evidence suggesting that endotoxin tolerance and immune homeostasis mechanisms may reduce sensitivity over prolonged exposure in pigs [12]. It is also demonstrated that low-dose chronic DON exposure modulated immune-relevant gene expression in pigs, with the magnitude of response diminishing over time, suggesting a form of adaptive tolerance [27].

It was documented that DON may induce oxidative stress and increase the release of reactive oxygen species, which result in damage to organs, including the liver and kidney, with subsequent changes in some enzymatic parameters and cells [1]. DON could cause severe liver damage through hepatocellular oxidative stress [29]. Several studies reported reduced serum superoxide dismutase and glutathione peroxidase, but increased serum MDA in weaning and immature gilts fed diets with 596–800 μg/kg DON [15,28]. Similarly, this study indicated that high-dose DON decreased serum T-AOC and LDH compared with low-dose DON in immature gilts. It was proposed that this may be due to the accumulated effects of DON. The exposure in the early stage did not reach the threshold. After 35 days of continuous feeding, DON triggered the long-term stress. During d 35–45, the gilts have adopted the DON exposure. Overall, the little impact of DON on serum profiles may mirror the results of growth performance in the present study.

Furthermore, the GnRH, LH, FSH, and E2 are key hormones of the reproductive axis, directly reflecting the secretion of gonadotropins and ovarian development in gilts [15]. It was observed that the serum reproductive hormones (GnRH, LH, FSH, or E2) did not differ among treatments, which was in line with a previous study. No difference was observed in serum FSH, LH, or E2 in immature gilts fed diets with 796 μg/kg DON [15]. The present results might indicate that DON has a relatively minor impact on the reproductive axis.

Combined with the results of different periods, it can be demonstrated that the DON did not exert a significant effect on the α-diversity and β-diversity of the fecal microorganisms in gilts in the current study. The relative abundance at the phylum level was mainly *Firmicutes*, *Bacteroidetes*, and *Spirales*, while the relative abundance of genera was *Lactobacillus*, *Streptococcus*, and *Clostridium*. The high-dose DON group decreased the abundance of *Streptococcus*. This may be due to the lack of DON effects on intestinal morphology and integrity in pigs [10,13,30]. Furthermore, DON, as a protein synthesis inhibitor, may exert direct antimicrobial activity against certain bacterial taxa. A recent report demonstrated that DON can selectively impair the growth of Gram-positive bacteria in the gut microbiota, including *Streptococcus* species, through its inhibition of bacterial ribosomal function [31]. It is reviewed that mycotoxins can directly modulate gut microbial composition through both antimicrobial activity and alteration of the gut microenvironment [32]. In addition, the *Streptococcus* species interact with host immunity [28]. A decrease in *Streptococcus* may mirror the changes in serum immunity indicators in this study.

The present study design compared two naturally occurring DON levels without including a zero-DON control group. While this limits our ability to quantify the absolute effect of DON, it reflects the practical reality of commercial pig production. Future studies incorporating a clean control alongside graded DON concentrations would provide a more complete dose–response characterization.

## 5. Conclusions

Based on the results of the present study, it may be inferred that dietary exposure to 1223 μg DON/kg tended to adversely affect serum inflammatory parameters and immune indices in immature gilts, whereas no statistically significant influence on growth performance was observed under the current experimental conditions. It emphasized that while the current limits are protective against overt toxicity, the immunological effects observed at 1223 μg/kg highlight the importance of maintaining strict DON controls in gilt and breeding sow diets.

## Figures and Tables

**Table 1:** Diet composition (as-fed basis).

Items	Percentage, %
Ingredients, %	
Corn	56.60
Soybean meal, 43%	10.20
Barley	20.00
DDGS	10.00
Limestone	1.20
Lysine (70%)	0.66
Sodium chloride	0.45
Monocalcium phosphate	0.33
Threonine (99%)	0.10
Methionine (99%)	0.10
L-tryptophan (98.5%)	0.13
Complex enzyme	0.03
Premix ^1^	0.20
Total	100.00
Analyzed value	
CP, %	13.7
DE, kacl/kg ^2^	3330
SID lys, %	0.90
SID Met,%	0.30
SID(Met + Cys), %	0.50
SIDThr, %	0.52
SIDTrp, %	0.15
Ca, %	0.60
P, %	0.42

^1^ Provided per kilograms of diet: vitamin A, 17,500 IU; vitamin D_3_, 5000 IU; vitamin E, 40 IU; vitamin K_3_, 5 mg; vitamin B_1_, 5 mg; vitamin B_2_, 12.5 mg; vitamin B_6_, 7.5 mg; vitamin B_12_, 0.05 mg; biotin, 0.2 mg; folic acid, 2 mg; niacin, 30 mg; D-calcium pantothenate, 25 mg; Fe, 100 mg as ferrous sulfate; Cu, 17 mg as copper sulfate; Mn, 40 mg as manganese oxide; Zn, 100 mg as zinc sulfate; I, 0.25 mg as potassium iodide; and Se, 0.25 mg as sodium selenite. ^2^ Calculated values.

**Table 2:** Effects of different levels of DON on the relative abundance of fecal microbiota at the phylum level in immature gilts ^1,2^.

Species Name	LD	HD	*p*_Value
*Firmicutes*	73.18 ± 16.43	72.52 ± 15.58	0.7016
*Bacteroidota*	13.15 ± 9.09	14.48 ± 9.52	0.4501
*Proteobacteria*	6.73 ± 16.82	4.57 ± 11.65	0.2828
*Spirochaetota*	2.99 ± 5.79	3.69 ± 5.37	0.2321
*Actinobacteriota*	1.39 ± 1.17	1.52 ± 1.62	0.5346
*Desulfobacterota*	0.99 ± 6.59	1.28 ± 7.91	0.1061
*Cyanobacteria*	0.25 ± 0.23	0.42 ± 1.09	0.4894

^1^ Means represent 6 replications (pens) per treatment (*n* = 6/group). ^2^ LD, low-dose 441 μg DON/kg diet; HD, high-dose 1223 μg DON/kg diet.

**Table 3:** Effects of different levels of DON on the relative abundance of fecal microbiota at the genus level in immature gilts ^1,2^.

Species Name	LD	HD	*p*_Value
*Streptococcus*	8.159 ± 7.365	4.705 ± 4.585	0.0117
*norank_f_Peptococcaceae*	0.04602 ± 0.03886	0.0735 ± 0.07409	0.0245
*Eubacterium_brachy_group*	0.01913 ± 0.04953	0.02203 ± 0.0265	0.0451
*Microbacterium*	0.00957 ± 0.04216	0.00103 ± 0.00399	0.0447
*Bacteroides_pectinophilus_group*	0.00335 ± 0.00729	0.00165 ± 0.00606	0.0475
*Mesorhizobium*	0.00377 ± 0.00992	0.00014 ± 0.00073	0.0204

^1^ Means represent 6 replications (pens) per treatment (*n* = 6/group). ^2^ LD, low-dose 441 μg DON/kg diet; HD, high-dose 1223 μg DON/kg diet.

## Data Availability

The data that support the findings of this study are available from the corresponding author upon reasonable request.
